# Sport- und Bewegungstherapie bei Burn-out und Fatigue – eine narrative Übersichtsarbeit

**DOI:** 10.1007/s00103-024-03967-6

**Published:** 2024-10-31

**Authors:** Lars Gerland, Freerk Theeagnus Baumann

**Affiliations:** 1 Institut für Kreislaufforschung und Sportmedizin, Abteilung II: Molekulare und zelluläre Sportmedizin, Deutsche Sporthochschule, Köln, Deutschland; 2 AG Onkologische Bewegungsmedizin/Klinik I für Innere Medizin, Centrum für Integrierte Onkologie Aachen Bonn Köln Düsseldorf, Universität zu Köln, Köln, Deutschland

**Keywords:** Bewegungstherapie, Fatigue, Körperliche Aktivität, Burn-out, Rehabilitation, Exercise therapy, Fatigue, Physical activity, Burnout, Rehabilitation

## Abstract

Burn-out und Fatigue weisen in ihren Symptomausprägungen Schnittmengen auf. Der gemeinsame Nenner ist Erschöpfung. Körperliche Aktivität konnte teils als risikomindernder Faktor für die Entstehung bzw. die Ausprägung der Symptome nachgewiesen werden. Auch in der Akutphase, der Rehabilitation und Nachsorge von Burn-out sowie bei Erkrankungen und deren Behandlungen, die mit dem Auftreten von Fatigue assoziiert werden, gibt es Belege für einen Effekt von körperlicher Aktivität.

In der Burn-out-Forschung gilt physische Aktivität als risikomindernder Faktor und Coping-Strategie, konkrete Bewegungsempfehlungen in Bezug auf Symptomausprägungen existieren jedoch nicht. Im Bereich der Müdigkeit/Fatigue ist das Gesamtbild uneinheitlich: Für einzelne Krankheitsbilder gibt es bereits zielgerichtete Empfehlungen für die Bewegungstherapie im multimodalen Ansatz. So gibt es hohe Evidenzen für den Einsatz von angemessen dosierter körperlicher Aktivität bei Krebspatient:innen, die unter tumorassoziierter Fatigue (CrF) leiden, in der adjuvanten Therapie und Nachsorge. Andere mit dem Auftreten von Fatigue assoziierte Erkrankungen, beispielsweise Long und Post-Covid, sind diesbezüglich noch nicht ausreichend erforscht, um klare Aussagen zu einer Dosis-Wirkungs-Beziehung zu machen.

Diese Arbeit soll einen Überblick über den Stand der Bewegungsforschung bei Burn-out und Fatigue geben und so einerseits Therapieempfehlungen für die Behandelnden und die Patient:innen aussprechen, andererseits die Evidenzlage in denjenigen Teilbereichen beleuchten, in denen es (noch) keine allgemeinen und individualisierten Bewegungsempfehlungen gibt.

## Abstract (en)

Burnout and fatigue have overlaps in their symptoms. The common denominator is exhaustion. Physical activity has been shown to be a risk-reducing factor for the development or manifestation of symptoms. There is also evidence of an effect of physical activity in the acute phase, rehabilitation, and aftercare of burnout as well as in diseases that are associated with the occurrence of fatigue and their treatments.

In burnout research, physical activity is considered a risk-reducing factor and coping strategy, but there are no specific exercise recommendations with regard to symptom severity. In the area of tiredness/fatigue, the overall picture is inconsistent: there are already targeted recommendations for exercise therapy in a multimodal approach for individual clinical pictures. For example, there is high evidence for the use of appropriately dosed physical activity in cancer patients in adjuvant therapy and aftercare. Other diseases associated with the occurrence of fatigue, such as long- and post-COVID, have not yet been sufficiently researched to make clear statements about a dose–response relationship. For some diseases, there are already targeted recommendations for exercise therapy in a multimodal approach.

This paper aims to provide an overview of the current state of exercise research in burnout and fatigue and thus, on the one hand, make therapy recommendations for practitioners and patients, and on the other hand, shed light on the evidence in those areas in which there are (still) no general and individualized exercise recommendations and guidelines.

## Hintergrund

Burn-out und Fatigue werden zwar auf fachlicher Ebene voneinander abgegrenzt, weisen jedoch in Bezug auf die Symptomausprägungen viele Gemeinsamkeiten auf. Schnittmengen finden sich auf emotionaler, kognitiver und physischer Ebene. In Tab. 1 werden die Symptome der Syndrome Müdigkeit/Fatigue nach der S3-Leitlinie „Müdigkeit“ [1] und Burn-out nach der Definitionszusammenstellung von Schaufeli und Enzmann [2] gegenübergestellt. Der gemeinsame Nenner der Vielzahl an Symptomen ist Erschöpfung.

**Tab. 1:** Symptomgegenüberstellung Burn-out und Fatigue nach den Definitionen der S3-Leitlinie „Müdigkeit“ [1] sowie Schaufeli und Enzmann [2]

Symptomqualität	Müdigkeit/Fatigue	Burn-out
Emotionale Aspekte	Unlust Motivationsmangel Enge Verbindung zu Traurigkeit Niedergedrückte Stimmung Verminderte affektive Schwingungsfähigkeit	Niedergeschlagenheit Begeisterungsverlust Reizbarkeit Desinteresse Arbeitsunzufriedenheit Niedrige Moral
Kognitive Aspekte	Verminderte geistige Aktivität Brain Fog (Bewusstseinstrübung)	Hilflosigkeit Zynismus Gefühl fehlender Anerkennung
Verhaltensaspekte	Leistungsknick	Hyperaktivität Aggressivität Reduzierte Effektivität
Körperliche Aspekte	Muskuläre Schwäche	Kopfschmerzen

Beide Themenbereiche und die jeweiligen Behandlungsmethoden sind noch jung. Neue Krankheiten wie etwa COVID-19 führen zu zusätzlicher Dynamik in der Forschung. In einigen Teilbereichen ist die Studienlage zum Einfluss von Sport- und Bewegungstherapie bei Burn-out und Fatigue noch dünn, in anderen Sektoren, beispielsweise in der Onkologie, hat sie schon klare Bewegungsempfehlungen in Abhängigkeit von der Symptomausprägung hervorgebracht.

Ziel dieses Reviews ist es, eine Übersicht über den aktuellen Stand der Forschung zum Thema Sport- und Bewegungstherapie bei Burn-out und bei Fatigue zu liefern, jeweils für die Prävention und die Akut- und Rehabilitationsphase. Die Abgrenzung zwischen Akut- und Rehabilitationsphase ist bei Burn-out und Fatigue kaum möglich, da die beiden Syndrome auch im Rahmen von Behandlung, Rehabilitation oder Nachsorge auftreten können. Die Übersichtsarbeit führt neben Leitlinien, systematischen Reviews und Metaanalysen bei Themenbereichen mit geringer Studienanzahl auch Einzelstudien auf.

## Burn-out und Wirkung bewegungstherapeutischer Interventionen

Der Begriff „Burn-out“ hat mittlerweile Einzug in die Umgangssprache gehalten, fernab von diagnostizierten Gesundheitsstörungen. Die enorme Vielfalt des Erscheinungsbildes erschwert die Abgrenzung, ebenso die Art und Intensität der Symptomausprägungen. Allgemein bezeichnet das Syndrom einen Zustand der physischen und mentalen Erschöpfung. Burn-out ist keine anerkannte somatische oder psychiatrische Krankheit, hat aber Krankheitswert (Diagnoseziffer Z73.0 ICD-10 „Probleme mit der Lebensführung“; [3]). Die 3 primären Symptome sind: emotionale Erschöpfung, Depersonalisation und reduzierte persönliche Leistungsfähigkeit [4].

In vielen Berufen sind Arbeitnehmer zunehmend von Burn-out betroffen, häufiger Frauen und ältere Menschen. Die Kosten für Arbeitgeber und Krankenkassen sowie die volkswirtschaftlichen Produktionsausfälle des Jahres 2020 aufgrund psychischer Erkrankungen – darunter Burn-outs – werden mit 14,6 Mrd. € angegeben [5]. Die Fertigstellung der S3-Leitlinie „Prävention des Burnouts“ ist für März 2026 geplant.

Insbesondere helfende Berufe waren schon frühzeitig Gegenstand der Burn-out-Forschung, Prävalenz und Symptomausprägungen sind gut dokumentiert. Beispielsweise können Onkolog:innen von Burn-out betroffen sein. In ihrer täglichen Arbeit mit Entscheidungen über Leben und Tod konfrontiert, müssen sie über hochtoxische und teure Behandlungen entscheiden und die Kommunikation mit Patient:innen und Angehörigen auch in Palliativsituationen meistern. Sie leiden in diesem anspruchsvollen Umfeld häufig unter Stress, Depressionen, Angstzuständen, Fatigue, einer verminderten Lebensqualität und unter Burn-out. 35 % der medizinischen Onkolog:innen, 38 % der Strahlentherapeut:innen und 28–36 % der onkologischen Chirurg:innen berichten über Burn-out-Symptome [6]. Die Beeinträchtigungen können sich negativ auf die Behandlung der Tumorpatient:innen auswirken, aber auch profunde Auswirkungen auf die Onkolog:innen selbst haben bis hin zu suizidalen Gedanken. Auch Medizinstudent:innen und Auszubildende in medizinischen Berufen leiden häufiger an Burn-out als die sonstige Bevölkerung [7].

Da auch die Entstehung eines Burn-outs in der subjektiven Wahrnehmung schleichend sein kann, ist wiederum die Abgrenzung von Prävention und Akutphase in der Studienlage mitunter schwer. Vor allem Querschnittsstudien zum Thema Burn-out und körperliche Aktivität unterscheiden teilweise nicht zwischen der Prävention und der Symptomminderung eines bestehenden Burn-outs [3]. Zur besseren Übersicht soll in dieser Arbeit eine Abgrenzung versucht werden.

### Prävention.

In ihrem systematischen Review mit 21 Studien legen Mincarone et al. [8] dar, dass physische Aktivität mit einem reduzierten Burn-out-Risiko einhergeht, insbesondere in den Domänen emotionale Erschöpfung und Depersonalisierung.

In einem weiteren systematischen Review fanden Taylor et al. [9] 18 Studien zum Einfluss von körperlicher Aktivität auf Burn-out und die Lebensqualität von Medizinstudent:innen. Diese belegten den positiven Einfluss von Training auf die Lebensqualität und eine Verminderung der Burn-out-Prävalenz. Die untersuchten Studien nutzten unterschiedliche Methoden, in erster Linie Fragebögen, zur Ermittlung des Umfangs körperlicher Aktivität. Sie gingen nur teilweise auf die Art der Bewegung und die Intensitätsniveaus ein.

Die im Review von Taylor et al. inkludierte Querschnittsstudie von Dyrbye et al. ([10]; *n* = 4402) differenzierte zwischen Ausdauerbelastung mit moderater oder hoher Intensität sowie Krafttraining. Die Analyse deutet darauf hin, dass höhere Trainingsintensitäten und -frequenz höhere Effektstärken hervorbrachten, fand jedoch keinen signifikanten Unterschied zwischen Ausdauertraining, Krafttraining oder der Kombination aus beiden.

Allgemein bewirkt ein höheres Niveau an körperlicher Aktivität einen moderaten Schutz vor dem Auftreten von Burn-out-Anzeichen, wenngleich die Wirkmechanismen nicht klar sind. Probleme in der Studienlage liegen in den unterschiedlichen Assessmentverfahren zu Burn-out. Das oftmals verwendete Maslach Burnout Inventory (MBI) ist nicht klinisch validiert. Die Autoren weisen selbst darauf hin, dass das MBI nicht als Werkzeug zur Diagnose oder als Indikator für die Notwendigkeit einer Intervention genutzt werden sollte [11]. Auch zu Art, Umfang und Intensität der körperlichen Aktivität sind nicht in allen vorliegenden Studien klare Aussagen getroffen worden. Korczak et al. kritisieren den Mangel an systematischen Wirksamkeitsstudien im Hinblick auf Burn-out-spezifische Prävention [12].

### Akutphase/Rehabilitation.

In einem systematischen Review zu Coping-Strategien von Mitarbeitern im Gesundheitssektor mit Burn-out identifizierten Maresca et al. [13] 7 Studien, deren Fokus auf dem Umgang mit den Symptomen vorliegender Burn-out-Fälle lag. Die Studien nutzten neben dem MBI auch andere Fragebögen sowie offene Interviews. In 2 Studien wurde körperliche Aktivität von Probanden als Coping-Strategie angegeben. Über die Art und den Umfang der körperlichen Aktivität wurde dabei keine Aussage getroffen.

Naczenski et al. [14] fanden moderate Evidenz für einen positiven Einfluss von körperlicher Aktivität auf Burn-out-bedingte Erschöpfung in 4 Längsschnittstudien sowie starke Evidenz in 6 Interventionsstudien.

Eine jüngere Studie, die nicht in den oben aufgeführten Reviews gelistet ist, belegt, dass auch moderate Level körperlicher Aktivität bereits eine effektive Senkung auf der Subskala emotionale Erschöpfung und eine verbesserte persönliche Zielrealisierung bewirken [15]. Die Subgruppen mit moderater und hoher körperlicher Aktivität schnitten zudem im Bereich der eigenen Leistungseinschätzung signifikant besser ab als die Subgruppe geringe körperliche Aktivität.

Trotz vielversprechender Studien zum Einfluss der körperlichen Aktivität auf Burn-out-Symptomatiken ist ein Mangel an Untersuchungen zu optimaler Intensität, Frequenz, Umfang und Art des Trainings festzustellen [9, 14]. Außerdem besteht auch in diesem Fall die Problematik der Definition einzelner Burn-out-Subskalen und der Parameter körperlicher Aktivität, die die Bestimmung einer Dosis-Wirkungs-Beziehung zusätzlich erschweren.

## Fatigue und Wirkung bewegungstherapeutischer Interventionen

Fatigue beschreibt eine signifikante Müdigkeit und Erschöpfung [1]. Oftmals sind das Auftreten und die Ausprägung unabhängig von vorangegangener Belastung körperlicher oder geistiger Art. Sie wird häufig im Kontext mit bestimmten Krankheiten und deren Therapien diagnostiziert. Während und nach der Corona-Pandemie kam das Thema Erschöpfung/Fatigue im Zusammenhang mit dem Post-Covid-Syndrom in den öffentlichen Fokus, doch auch bei chronischen Erkrankungen, bei Viruserkrankungen und Krebserkrankungen und deren Therapien ist Fatigue ein häufiger Begleiter.

Die Internationale Klassifikation der Krankheiten ICD-10 beschreibt „Müdigkeit“ mit dem Code R 53. Das chronische Fatigue-Syndrom (CFS) und Encephalomyelopathie (ME) sowie postvirales Müdigkeitssyndrom werden mit G 93.3 codiert. Die Codes U 09.9! (Post-COVID-19-Zustand) und U10.9 (multisystemisches Entzündungssyndrom in Verbindung mit COVID-19) kamen in den vergangenen Jahren hinzu [1].

Im Zusammenhang mit Krebserkrankungen und -therapien auftretende Fatigue-Syndrome werden als tumorassoziierte Fatigue (Cancer-related Fatigue – CrF) bezeichnet. Es handelt sich um eine der häufigsten Nebenwirkungen, die mitunter schwer fassbar sind. Die klinischen Erscheinungsformen sind vielfältig und in der subjektiven Wahrnehmung höchst individuell. Müdigkeit, körperliche Schwäche und verminderte Leistungsfähigkeit treten auch ein Jahr nach Therapieabschluss bei etwa einem Drittel der Patient:innen auf und persistieren bei 20 % der Patient:innen mehrere Jahre nach Abschluss der Behandlung [16].

Fatigue gilt bei COVID-19 und Post-Covid-Erkrankungen als größter Einflussfaktor auf die Lebensqualität [17]. 12,82 % der Probanden einer großen Kohortenstudie (*n* = 273.618) gaben im Zeitraum 1–180 Tage nach der Erkrankung Fatigue-Symptome an. Im Zeitintervall 90–180 Tage waren es noch 5,87 % [18]. 6 Monate nach stationärer Behandlung gaben 63 % der Teilnehmer einer Verlaufsstudie Fatigue oder Muskelschwäche an [19]. Gemäß der S1-Leitlinie Long/Post-COVID [20] muss allerdings ergänzt werden, dass ein Kausalzusammenhang zwischen der Erkrankung und Fatigue-Symptomen nicht zwingend vorliegt.

Auch wenn es auf den ersten Blick widersprüchlich anmutet, Patient:innen mit Fatigue-Symptomen zu körperlicher Aktivität zu ermutigen, gibt es in den verschiedenen Kontexten bereits eindringliche Erfahrungen mit den Auswirkungen von Bewegung auf die Entstehung und das Management von Fatigue, die im Folgenden dargestellt werden solle.

### Prävention/Prähabilitation.

Bei der Erkrankung COVID-19 wurde physische Inaktivität mit einem 30 % höheren Risiko der Hospitalisierung verbunden [21]. Zudem ist mangelnde (respiratorische) Fitness ein unabhängiger Risikofaktor für die Überlebenswahrscheinlichkeit stationärer Akutpatient:innen [22]. Wir konnten keine Studie finden, die gezielt einen Einfluss des Aktivitätsniveaus auf die Prävalenz von Fatigue-Symptomen bei Covid- und Post-Covid-Patient:innen untersucht.

In der Onkologie gibt es darüber hinaus die Phase der Prähabilitation. Das Thema CrF ist hier jedoch noch wenig erforscht. Eine Interventionsstudie ([23]; *n* = 110) untersuchte den Einfluss von Fatigue-Schulung und -Support vor, während und nach der Radiotherapie, konnte aber zu keinem Messzeitpunkt signifikante Verbesserungen der Fatigue-Werte im Intergruppenvergleich feststellen. Durch die Maßnahme wurde allerdings die Partizipation an Bewegungsangeboten erhöht.

Segal et al. [24] konnten hingegen in einer Krafttrainingsstudie mit Prostatakrebspatienten (*n* = 155, Interventionsdauer: 12 Wochen) nachweisen, dass der frühzeitige Beginn einer Bewegungs‑/Trainingsintervention der Ausbildung von CrF vorbeugen kann. Die Effektstärke des Trainings in derselben Studie war zudem höher, als wenn bereits CrF-Symptome vorlagen. Die Wirksamkeit der Maßnahme, wenn sie erst ein Jahr nach Therapiebeginn aufgenommen wurde, war nur noch tendenziell festzustellen. Dies legt die Notwendigkeit der frühzeitigen Aufnahme von bewegungstherapeutischen Ansätzen nahe – zumindest bei Krebspatient:innen.

### Akutphase/Rehabilitation.

Während die Datenlage zur Prähabilitation bei *CrF* schwach ist, gibt es für die Akutphase und Nachsorge bereits umfassende Studien. In einer Metaanalyse von 2017 mit 113 randomisierten kontrollierten klinischen Studien wurden 3 verschiedene Interventionen zur Reduktion von CrF bei verschiedenen Krebsentitäten verglichen (11.525 erwachsene Probanden, 78 % Frauen, mittleres Alter 54 Jahre). Die Bewegungstherapie zeigte die höchste Effektstärke (0,30; 95 %-KI 0,25–0,36; *p* < 0,001) vor der Psychoonkologie (Effektstärke 0,27; 95 %-KI 0,21–0,33; *p* < 0,001) und der medikamentösen Therapie (Effektstärke 0,09; 95 %-KI 0,00–0,19; *p* < 0,05). Bewegungstherapie und Psychoonkologie wurden als wirksames Mittel zur Reduktion von CrF während der Krebstherapie eingestuft, einzeln und als Kombinationstherapie jeweils wirksamer als medikamentöse Behandlung [25]. Folgerichtig bewerten internationale Expertengruppen und Fachgesellschaften die Bewegungstherapie in Bezug auf Fatigue mittlerweile mit höchsten Evidenzgraden.

In der Frühphase der bewegungstherapeutischen Forschung stand zunächst das Ausdauertraining, mittlerweile gibt es jedoch eine ausreichende Studienlage zu verschiedenen Trainingsinterventionen, sodass spezifische Empfehlungen je nach Ausprägung der CrF gegeben werden können. Ausdauertraining gilt als sichere und wirksame Methode und ist vor allem bei denjenigen onkologischen Patient:innen anderen Trainingsinterventionen gegenüber zu bevorzugen, bei denen hohe Fatigue-Ausprägungen und ein erhöhtes Depressionsrisiko vorliegen [26]. Große Vorteile von Ausdauerinterventionen sind die geringen Einstiegshürden im Bereich Material, Supervision, aber auch Intensität, die sehr gut auf Patient:innen und deren Vorlieben zugeschnitten werden können.

Das Krafttraining mit Krebspatient:innen wurde in den vergangenen Jahren verstärkt in den Fokus der Wissenschaft gerückt. Die Exercise Guidelines von Campbell et al. [27] listen Krafttraining als effektive Maßnahme gegen CrF in der Akutphase. Krafttraining ist dabei zum Teil effektiver als andere Interventionen. So gab es in einem Intergruppenvergleich von Bestrahlungspatientinnen signifikante Intergruppenvergleiche zwischen Krafttraining und Entspannungsverfahren, wobei sich die erstgenannte Gruppe in höherem Maße verbesserte, insbesondere in den Parametern physische Fatigue [28, 29].

Noch im Detail zu ermitteln ist die tatsächliche Zielintensität des Krafttrainings, wie auch die Zyklisierung gerade in Hinblick auf Bestrahlungs- und Chemotherapietermine in der Akuttherapie. Eine Studie mit niedrigen Intensitäten konnte keinen Effekt von Krafttraining auf die Ausprägung von CrF belegen [30], wohingegen es positive Effekte in einer RC-Studie mit intensiverem Krafttraining im Vergleich zu einer Relaxationsgruppe gab [31].

2 Studien belegten auch langfristige positive Effekte auf subjektiv wahrgenommene CrF bei Probanden in progressiven Krafttrainingsprogrammen im Vergleich zur Kontrollgruppe [32, 33]. Dies sind wichtige Wegweiser für die langfristige Nachsorge von Tumorpatient:innen.

Einerseits liegt ein Wert von Bewegungsinterventionen bei CrF in der verhältnismäßig schnellen Messbarkeit ihrer Erfolge. Diese sind in der Regel schon 14 bis 21 Tage nach Beginn signifikant [34]. Daraus lässt sich schließen, dass entsprechende Bewegungsprogramme schon im stationären oder ambulanten Setting begonnen werden sollten, zugeschnitten auf die räumlichen Gegebenheiten und die Compliance der Patient:innen.

Andererseits können Tumorpatient:innen nach abgeschlossener medizinischer Therapie mit höheren Intensitäten trainieren, sodass die Maßnahmen im Interventionsverlauf durchaus unterschiedlich starke Effekte erzielen können [35]. Patient:innen unter Chemo- oder Strahlentherapie sollten dagegen in geringerem Maße belastet werden. Carayol et al. [36] zeigten bei Brustkrebspatientinnen in der adjuvanten Therapie die beste Wirksamkeit bei 4–5 h körperlicher Aktivität pro Woche bei moderaten Intensitätsniveaus.

Grundsätzlich lässt sich sagen, dass jede Form der körperlichen Aktivität sich positiv auf CrF auswirkt, die Auswahl der Intervention aber abhängig sein muss von der subjektiv empfundenen Fatigue, um sowohl die bestmögliche Effektivität als auch Compliance zu erreichen. Dabei sollte gerade in der Akuttherapie die Tagesform keinesfalls außer Acht gelassen werden. Mit einfachen Mitteln kann die Trainingssteuerung täglich individuell justiert werden. Visuelle Analogskalen oder die numerische Ordinalskala sind einfache Tools, mit deren Hilfe Patient:innen ihre CrF kurzfristig ausdrücken können. Vereinfacht gesagt, wird bei höher ausgeprägten CrF-Tageswerten mit niedriger Intensität trainiert, an besseren Tagen kann auch die Belastung durch die Bewegungsintervention höher ausfallen [37]. Die detaillierten Trainingsempfehlungen sind Tab. 2 zu entnehmen.

**Tab. 2:** Trainingssteuerung für Patient:innen mit Fatigue-Beschwerden; NAS = numerische Analogskala. (Mod. nach McNeely und Courneya [29])

Ausprägung Fatigue	Ausdauertraining	Krafttraining	Kommentare/Empfehlungen
Leichte Fatigue < 4/10 (NAS)	Progressives aerobes Ausdauerprogramm von 20–30 min. pro Session, 3–5 Tage/Woche, bei 60–80 % der max. Herzfrequenz	8–10 Übungen der großen Muskelgruppen der oberen und unteren Extremitäten sowie Rumpf; Widerstand: 60–70 % der max. Kraft; 8–12 Wiederholungen, 1–2 Sätze, 2–3 Tage/Woche	Bewegungsprogramm muss immer im multidisziplinären Ansatz erstellt werden Wichtig: Monitoring der Energiebilanz und damit auch der körperlichen Gesamtaktivität Berücksichtigung jeglicher akuter, chronischer und langfristiger Nebenwirkungen der Krebstherapie Stetige Überwachung der Fatigue-Symptome während und nach dem Bewegungsprogramm Bei moderater bis schwerer Fatigue: Mit Krafttraining beginnen um Muskelkraft und Kraftausdauer zu steigern Totale Erschöpfung vermeiden
Moderate Fatigue 4–6/10 (NAS)	Zunehmende Steigerung des Trainingsumfangs von 5–10 min pro Session; Steigerung, bis 60–80 % der max. Herzfrequenz erreicht sind; zunächst Trainingsdauer erhöhen, dann Intensität	8–10 Übungen der großen Muskelgruppen der oberen und unteren Extremitäten sowie Rumpf; Widerstand: geringe Intensität (z. B. 30–50 % der Maximalkraft; 10–15 Wiederholungen, 1–2 Sätze und zunehmend steigern zu 60–70 %)	
Starke Fatigue ≥ 7/10 (NAS)	Trainingseinheiten mit geringen Intensitäten, Walking/Radfahren von mehreren Einheiten á 5–10 min über den Tag verteilt, bis die empfohlene Gesamtdauer erreicht ist; Intervalltraining wird für diejenigen Patient:innen empfohlen, die nicht mit der Dauermethode trainieren können	Zunächst Training ohne Widerstand („active range of motion“) gegen die Schwerkraft; sofern toleriert, Addition kleiner Gewichte	

Im Bereich der viralen Erkrankungen hat *COVID-19* durch seine Neuheit und enorme Verbreitung einen großen Raum in der jüngsten Fatigue-Forschung eingenommen. Während der akuten Erkrankung sollte die körperliche Aktivität reduziert bleiben. Nach der Akutphase empfehlen Demeco et al. [38], von körperlicher Aktivität abzusehen, wenn (1) die Ruheherzfrequenz über 100 Schlägen/Minute liegt, (2) der Blutdruck unterhalb von 90/60 mm Hg oder über 140/90 mm Hg oder (3) die Blutsauerstoffsättigung unter 95 % liegt. Empfehlungen zu Umfang, Art und Intensität körperlicher Aktivität sind bisher rar und uneindeutig.

Auch bei der Therapie von Long Covid gibt es bisher keine evidenzbasierte oder gar allgemeine Empfehlung zur Symptombehandlung der betroffenen Patient:innen [39]. Mehrere Publikationen legen die Strategie nahe, bei Fatigue-dominanten Covid-Symptomatiken vor allem auf die Umsetzbarkeit der Maßnahmen und die Lebensqualität der Betroffenen abzuzielen [40]. Die aktuelle Empfehlung bei Long Covid lautet, dass Patient:innen nach eigenem Ermessen die Belastung steigern und bei einer Symptomverschlechterung die Intensität auf die vorangegangene Trainingsstufe reduzieren [40]. Die Überbeanspruchung mit möglicher nachfolgender Symptomverschlechterung (Post-exertional Malaise – PEM) soll gemäß der aktuellen S1-Leitlinie [20] durch wohldosierte und gegebenenfalls supervidierte körperliche Aktivität vermieden werden. Dabei steht insbesondere das individuell angemessene Energiemanagement (Pacing) im Vordergrund [41].

Die aktuellen Empfehlungen des Deutschen Verbands für Gesundheitssport und Sporttherapie e. V. (DVGS) zu Bewegung und körperlichem Training nach COVID-19 [41] sprechen sich bei bestehender Fatigue infolge einer COVID-19-Erkrankung für ein schrittweises Herantasten an die Bewegungsempfehlungen der Weltgesundheitsorganisation (WHO) aus, laut denen mindestens 150 min moderate Ausdaueraktivitäten pro Woche oder 75 min intensive Ausdaueraktivitäten pro Woche und zusätzlich kräftigende Aktivitäten für die großen Muskelgruppen an 2 Tagen pro Woche vorgeschlagen werden. Dabei sollen Patient:innen Pacing anwenden, ein individuelles „gesundes Mittelmaß“ finden. Zu diesem Zweck sind die Betroffenen angehalten, Bewegungseinheiten über den Tag und die Woche einzuteilen, Entspannungsphasen einzuplanen, Abwechslung bei den Aktivitäten zu schaffen und auf den eigenen Körper zu hören.

Da es sich bei COVID-19 um ein respiratorisches Virus handelt, liegt die Vermutung nahe, dass gerade Ausdauerbelastungen für die Patient:innen schnell zur Überlastung führen. RCTs zur Effizienz verschiedener Bewegungsprotokolle (z. B. Krafttraining, Kombinationstraining, Entspannungsverfahren) sind jedoch bisher nicht bekannt.

Konkrete Bewegungsempfehlungen gibt es demnach bislang nicht. Diejenigen Empfehlungen für Patient:innen, die lediglich auf subjektiver Einschätzung („Herantasten“) basieren, sind kritisch zu sehen, da schon die Selbsteinschätzung ein Grundmaß an sportlicher Vorerfahrung erfordert.

## Diskussion und Ausblick

Das Themenfeld der Bewegungstherapie bei Burn-out und Fatigue ist enorm breit gefächert, da sowohl die Erkrankungsformen als auch die Möglichkeiten der Bewegungstherapie äußerst heterogen sind.

Bei Burn-out sind insbesondere RC-Studien mit bewegungstherapeutischem Ansatz rar. Kohortenstudien und retrospektive Analysen kommen jedoch zum Schluss, dass körperliche Aktivität sowohl das Risiko des Burn-outs und seiner symptomatischen Manifestation reduziert als auch bei bereits bestehendem Burn-out eine Coping-Strategie für Patient:innen darstellt. Folgende Studien müssen jedoch zwingend mit systematischen Mitteln angemessene Dosis-Wirkungs-Beziehungen erforschen, um zielgerichtete Empfehlungen für Patient:innen auszusprechen. Auch das steigende öffentliche Bewusstsein für Burn-out kann so genutzt werden, wenn allgemeine Bewegungsempfehlungen für präventive körperliche Aktivität herausgearbeitet würden.

Aufgrund der Vielfältigkeit von Fatigue und ihren möglichen Ursachen können keine einheitlichen Therapieempfehlungen gegeben werden. So ist es beispielsweise bei akuten (respiratorischen) Viruserkrankungen kontraindiziert, zusätzliche physische Stressoren zu setzen. Bei Tumorpatient:innen hingegen mag es zwar ebenfalls widersprüchlich wirken, vor oder während der Therapie Bewegungstherapien zu implementieren, doch die Forschungslage empfiehlt genau dies – mit hohen Evidenzen.

Die Forschung im Bereich CrF ist allgemein so weit fortgeschritten, dass es konkrete Bewegungsempfehlungen für verschiedene Entitäten und Symptomausprägungen gibt. Diese Empfehlungen fehlen bei Viruserkrankungen und vielen anderen Fatigue-Syndromen. Die Basis der Richtlinien sind intensive RC-Studien mit verschiedenen Trainingsprotokollen, Settings, guter Trainingssteuerung und aufwendigen Testverfahren. Wenngleich es auch im Bereich CrF noch viele unterrepräsentierte Interventionsmöglichkeiten in der Bewegungsforschung gibt, kann die Forschung der letzten Jahrzehnte zum Vorbild genügen mit dem Ziel, für jeden Patienten und jede Patientin eine individuelle, sichere, effektive und motivierende Intervention empfehlen zu können.

Im Bereich von COVID-19, Long und Post-Covid sind die Empfehlungen limitiert durch die schwache Studienlage, die sich wiederum aus der Novität der Erkrankung ergibt. In der Akutphase sind Bewegungsinterventionen kontraindiziert, in der Long und Post-Covid-Phase ist der Kernpunkt der Bewegungsempfehlung das Pacing, das individuelle Herantasten an physische Belastung durch Bewegung und Training.

**Abb. 1:**
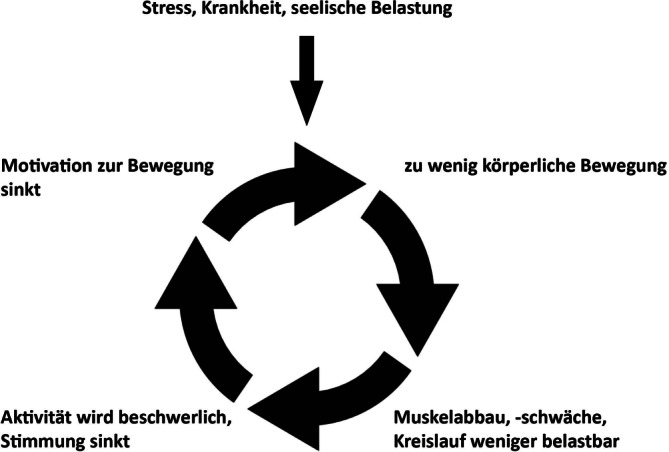
Kreislauf in der Entwicklung von Müdigkeit nach S3-Leitlinie „Müdigkeit“

Die heterogenen Kollektive der genannten Syndrome lassen allgemeine Bewegungsempfehlungen nicht zu. Jedoch kann die Aussage getroffen werden, dass durch körperliche Aktivität die Abwärtsspirale der Erschöpfung [1] unterbrochen werden kann (Abb. 1).

## Fazit

Die Sport- und Bewegungstherapie ist eine vergleichsweise kostengünstige, vielfältige und motivierende Intervention, die in ihrer Art und Ausprägung stark an die Patientin oder den Patienten angepasst werden kann. Bewegungsprogramme können stationär, in der Rehabilitation, aber auch in heimbasierten Programmen durchgeführt werden. Videokurse, Apps oder Informationsbroschüren können dabei Versorgungslücken füllen, wenn entsprechende Strukturen (Gruppen, Trainingsräume, Therapeut:innen) nicht vorliegen. Die Vielfalt der Sportangebote ermöglicht es zudem, neben der Effizienz auch die persönlichen Vorlieben der Patient:innen zu berücksichtigen und so ein hohes Motivationsniveau sicherzustellen.

Die aktuellen Empfehlungen bei turmorassoziierter Fatigue in der Rehabilitation bilden ein Netz aus psychosozialen und bewegungstherapeutischen Ansätzen, das auf die individuellen Beeinträchtigungen zugeschnitten werden kann. Bewegungstherapie und psychoonkologische Interventionen sollten als Erstlinientherapie bei der CrF verschrieben werden [24].

Die Bewegungstherapie nimmt einen wichtigen Platz im multidisziplinären Behandlungsansatz ein. Um diesen Platz bei sämtlichen Fatigue-Symptomatiken und in der Prävention und Behandlung von Burn-out einzunehmen, sind die genannten Forschungsansätze mit hoher Dringlichkeit zu verfolgen.
